# The Study of an Adaptive Bread Maker Using Machine Learning

**DOI:** 10.3390/foods12224160

**Published:** 2023-11-17

**Authors:** Jooho Lee, Youngjin Kim, Sangoh Kim

**Affiliations:** Department of Plant and Food Engineering, Sangmyung University, Cheonan 31066, Republic of Korea; 201920724@sangmyung.kr (J.L.); 201820720@sangmyung.kr (Y.K.)

**Keywords:** bread maker, baking process prediction, artificial intelligence, machine learning, computer vision

## Abstract

Bread is one of the most consumed foods in the world, and modern food processing technologies using artificial intelligence are crucial in providing quality control and optimization of food products. An integrated solution of sensor data and machine learning technology was determined to be appropriate for identifying real-time changing environmental variables and various influences in the baking process. In this study, the Baking Process Prediction Model (BPPM) created by data-based machine learning showed excellent performance in monitoring and analyzing real-time sensor and vision data in the baking process to predict the baking stages by itself. It also has the advantage of improving the quality of bread. The volumes of bread made using BPPM were 127.54 ± 2.54, 413.49 ± 2.59, 679.96 ± 1.90, 875.79 ± 2.46, and 1260.70 ± 3.13, respectively, which were relatively larger than those made with fixed baking time (*p* < 0.05). The developed system is evaluated to have great potential to improve precision and efficiency in the food production and processing industry. This study is expected to lay the foundation for the future development of artificial intelligence and the food industry.

## 1. Introduction

Bread, a food made from simple ingredients such as flour, water, salt, and yeast, is one of the most consumed foods in the world and provides micronutrients and minerals such as Fe, K, Mg, Ca, Cu, and Zn, compounds that interact with immune cells to help the body grow, develop, and maintain itself [1,2]. Bread is a flavorful, nutrient-rich food that has been around since the Neolithic period around 10,000 B.C. and continues to be a staple of the diet to this day. The first bread in a form similar to today’s was found in Egypt, and it is documented that the reason the construction of the pyramids was delayed was due to a lack of an adequate supply of bread [3]. Bread has been made by humans for thousands of years and has been adapted to the basic qualities of the raw materials, the culture, and the nature of the society. Despite its long history in various countries, bread continues to be consumed and is considered a staple food in many countries [4]. In particular, white bread can influence consumer preference due to its shape, texture, and flavor, and is more often consumed as a meal replacement due to its even nutrient composition [5]. Bread continues to be highly consumed regardless of the season, and with the growing interest in healthier foods, bakers are introducing breads with healthier ingredients such as whole grain rye, brown rice, barley, and whole wheat rather than traditional ingredients [6].

In line with this trend, research is actively being conducted to analyze the properties of wheat flour by adding other grains or functional ingredients in addition to wheat flour [7]. Lightwave ovens, which use the principle of electrical resistance to initiate heating and bake bread based on the flow of electric current, vary in durability, efficiency, and availability from product to product, so environmentally variable values such as latency and temperature may not be up to standard [8]. For these reasons, product quality can change suddenly during production, so a reliable strategy is needed to monitor the manufacturing process. Therefore, the focus is on the advancement of system digitalization; the introduction of modern machines and tools; social, economic, and environmental issues, thus reducing process costs; minimizing the use of water, fuel, and fertilizers; and promoting the use of renewable energy. The use of Wireless Sensor Networks (WSN) has been investigated to compensate for these problems [9]. WSN is one of the most important technologies of the 21st century and a core technology of the Internet of Things, which has fundamentally changed human life [10]. WSN can be defined as a low-cost platform for connecting large-scale sensor networks in that it is cheaper, smaller, and can include a larger number of sensors compared to traditional wired sensor networks [11]. In a prior study that applied WSN to the food industry, Wang et al. (2015) developed a WSN-based food supply chain monitoring system and improved methods to prevent the transport of perishable foods in real time [12]. Therefore, digital monitoring to meet customer needs provides important knowledge about the production stage and can optimize product quality by detecting weaknesses in the overall process [13]. Artificial intelligence (AI) is a critical element of the important fourth industrial revolution, which is improving research in engineering, science, medicine, food and nutrition, marketing, stocks, and a wide range of fields [14]. Machine learning (ML) is a subfield of AI that uses a wide range of statistical techniques in which computer programs learn to associate data with predictive power.

AI and ML have actually been used in the food industry for food quality control, food science and processing, and wine analysis [15,16]. In the food processing field, Du et al. (2005) [17] classified pizza shapes and toppings using an image processing system. In a food quality assessment study, Perrot et al. (1996) [18] used sensor fusion to evaluate the optimal endpoint of cookie baking. Kim (2022) [19] used ML with a multilayer perceptron to predict the electrical energy of freezers and optimize electrical energy consumption. Gonzalez Viejo et al. (2022) [20] evaluated the quality characteristics of sourdough bread using digital technology and ML modeling. Additionally, Golcuk et al. (2023) [21] classified bread wheat genotypes using an ML algorithm. As such, AI and ML are increasingly being applied in the food industry to predict and evaluate the information and quality characteristics of food. Although there are various studies using AI in food processing, there is little research on applying AI and ML to the bread-making process to predict and control the baking process. Therefore, in this study, a data acquisition device that collects sensor and vision data was developed to determine the impact of various variables such as product characteristics, environmental conditions, and baking location on the baking process. Ultimately, the bread maker with AI developed in this study aims to realize a bread expert system based on sensor and vision data by predicting the baking stages by itself, unlike bread made by an automatic machine with fixed baking time and temperature.

## 2. Materials and Methods

### 2.1. Materials

The following ingredients were used to make the bread in this experiment: strong flour (CJ Cheiljedang Co., Ltd., Seoul, Republic of Korea), instant dry yeast (EverHealthCare Co., Ltd., Icheon-si, Republic of Korea), sugar (Beksul Co., Ltd., Seoul, Republic of Korea), cooking oil (Sajo Co., Ltd., Anseong-si, Republic of Korea), and distilled water extracted by a water purification system (FTPF09550; Merck Millipore Corp., Darmstadt, Germany).

### 2.2. Methods

#### 2.2.1. Baking Method

The bread for obtaining sensor and vision data was made according to AACC Method 10-10B [22], and the flour was weighed using an electronic scale (SW-1S; CAS Corporation, Yangju-si, Republic of Korea). The amounts of the remaining ingredients were weighed using a precision scale (GB303; Mettler Toledo Inc., Greifensee, Switzerland). For the bread, the amount of each ingredient was set according to the method of You et al. (2021) [23]. The control group (CBR1-5) is automatically made according to the proportions in Table 1 based on the weight of flour and the preset kneading, fermentation, and baking temperatures and times in a multifunctional bread maker (KBM-1100B; Jiangmen Mielux intelligent and Technology Co., Ltd., Guangdong, China). The weighed ingredients were put into the dough tub of the multifunctional bread maker. 

The materials of experimental group (EBR1-5) are identical to those in the control group, as shown in Table 1. The experimental group was made by modifying a multifunctional bread maker, and the device control was achieved through an artificial intelligence model generated by ML based on data from experiments of control group.

#### 2.2.2. Construction of the Baking Blackout Chamber (BBC)

**Figure 1:**
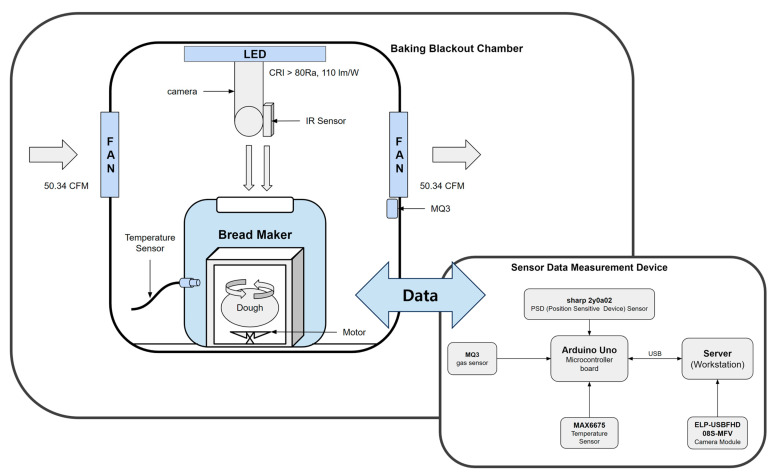
Configuration of Baking Blackout Chamber (BBC) with Sensor Data Measurement Device (SDMD).

The Sensor Data Measurement Device (SDMD) is configured with the BBC, as shown in Figure 1. External light is blocked by the BBC. For smooth heat dissipation during the baking stage, two fans (F129025SH; Everflowtech Corp., New Taipei City, Taiwan) for intake and exhaust were installed in front and behind the BBC to achieve an air volume of 50.34 CFM × 2. An LED lamp (LM180180M15; Lumenlux Co., Bucheon-si, Republic of Korea) was installed above the BBC to achieve a color rendering of CRI > 80 Ra and a light efficiency of 110 lm/W for uniform brightness. Workstation can communicate with the SDMD and is configured to store sensor and vision data from the SDMD.

#### 2.2.3. Collect Device Data for ML

The raw data of the baking process was measured by the SDMD, as shown in Figure 1, to generate the dataset needed to predict the baking process. The SDMD was designed and built based on an Arduino (Uno, Arduino Co., New York, NY, USA) and configured to collect sensor and vision data from inside the bread maker dough tub throughout the baking process. The SDMD consists of a K-type thermocouple module MAX6675 (SZH-CH031, Analog Devices Inc., Wilmington, MA, USA), a gas sensor MQ-3 (SEN040411, Henan Hanwei Electronics Co., Ltd., Zhengzhou, China), and a Position-sensitive Device (PSD) sensor (GP2Y0A02YK0F, Sharp Microelectronics Corp., New York, NY, USA), each connected to a workstation via USB. A high-speed camera (ELP-USBFHD08S-MFV, Shenzhen Ailipu Technology Co., Ltd., Shenzhen, China) is connected to the workstation via USB and measures vision data. 

The camera is configured to collect real-time vision data of bread during the baking process using the computer vision library OpenCV. OpenCV is an open-source library that is primarily used for computer vision applications [24]. The contours of the bread were detected after converting RGB, OpenCV’s existing color format, to grayscale. A Gaussian function was applied to remove noise and improve image sharpness. In addition, the closing operation of the shape calculation, which applies a dilation operation followed by an erosion operation, was performed to smooth the contours. Table 2 shows the measurements and performance of each sensor configured in the SDMD. Sensor and vision data are measured by the SDMD from the time the bread maker finishes kneading and switches to fermentation mode. 

The operation settings of the multifunctional bread maker used in the control group’s experiments were analyzed, and the results showed that fermentation is carried out at a temperature of 37 ± 3 °C in fermentation mode, and baking is carried out at a temperature of 165 ± 3 °C in baking mode after the fermentation mode is completed. The data collected by SDMD in this process resulted in 19,170 fermentation data sets and 14,524 baking data sets. The data sets consist of temperature, ethanol, distance, RGB, radius, and SPGV (Sum of Grayscale Value). The data sets were analyzed and labeled as fermentation and baking modes. The data sets were randomly split 8:2 between training data and validation data.

#### 2.2.4. Data Preprocessing Methods

Experiments were conducted on a workstation (ideaPad Gaming 3 15IHU6, Lenovo Group Ltd., Beijing, China). The CPU is 11th Gen Intel(R) Core(TM) i5-11300H, the GPU is NVIDIA GeForce RTX 3050 Laptop GPU, RAM is 32.0 GB, 64-bit Windows 11 22H2, and the programming language is Python (Version 3.11.4). Python is an advanced programming language introduced in 1991 by Dutch software engineer Guido van Rossum. It is a platform-independent, object-oriented, dynamically typed, and interactive language [19]. Scikit-learn (Version 1.3.0), an open-source library that provides Python-based supervised and unsupervised learning algorithms, was used for the prediction models. Normalization is a technique to ensure that sensor data in a database have similar ranges. This is very important when the data are unstructured and contain outliers. MinMaxScaler normalization is advantageous for high-dimensional data [25]. The following expression shows the MinMaxScaler normalization method:

(1) $$X_{std}=\frac{\left(X-X.\;min\right)}{\left(X.max-X.min\right)}$$

(2) $$X_{scaled}=X_{std}\times (X.max-X.min)+X.min$$

For the training dataset, the MinMaxScaler normalization method was used to scale the input data temperature, ethanol, distance, RGB, radius, and SPGV and stored in a csv file. 

#### 2.2.5. Baking Process Prediction Model (BPPM)

**Figure 2:**
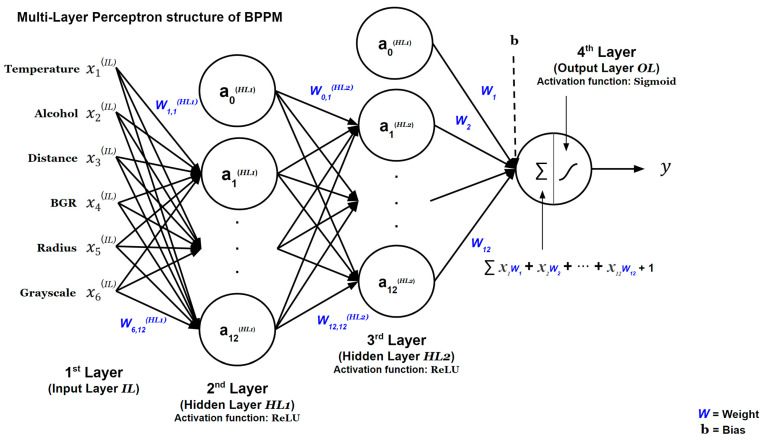
Multilayer perceptron (MLP) structure of BPPM.

The multilayer perceptron (MLP) structure of BPPM is shown in Figure 2. The open-source software libraries Tensorflow (Version 2.13.0) and Keras (Version 2.13.1) were used to utilize ML with MLP. Data-based BPPM is a binary classification sequential model that classifies the given input data into two labels. The model, labeled stay mode and stage transition, was configured with a threshold of 0.85, such that exceeding the threshold would result in a stage transition from stay mode. 

In BPPM, the Fermentation Stage Prediction Model (FSPM) and Baking Stage Prediction Model (BSPM) are stored in pkl format, and the Input Layer (IL) of each BPPM model is input to the first neural network Hidden Layer 1 (HL1). Twelve hidden units were used in HL1, and the Rectified Linear Unit (ReLU) activation function was used. The second Hidden Layer 2 (HL2) also has 12 hidden units, similar to HL1, and uses the ReLU activation function. The last layer, the Output Layer (OL), uses the sigmoid activation function, which outputs values between 0 and 1 and is suitable for binary classification. Sigmoid functions are widely used as activation functions in neural networks due to their bipolar transmission properties [26]. The logistic function, an example of a sigmoid, can be expressed as follows:

(3) $$logistic:\;f\left(x\right)=\frac{e^{x}}{e^{x}+1}$$

In OL, the sum of the input data and bias is multiplied by the weight, and the sum of the values is input into the sigmoid activation function to calculate the result. As a loss function, binary cross entropy is used to calculate the difference between the predicted and actual values output by the sigmoid activation function, and Adaptive Moment Estimation (ADAM) is used as an optimizer. Each BPPM was trained on a training dataset with a batch size of 1024 for a total of 1000 epochs, and its accuracy and loss were measured on the evaluation data. A manual search method was used to optimize the hyperparameters. The manual search method refers to the process where the researcher manually selects the hyperparameters to be evaluated. This method can quickly arrive at a reasonable solution based on intuition about the importance of various hyperparameters [27].

#### 2.2.6. Measurement of the Volume and Specific Volume of Bread

The bread made by the bread maker was cooled, packaged, and preserved at 25 °C for 24 h, after which the volume of the bread was determined by the rapeseed displacement according to AACC Method 10.05-01 [22]. After filling the 335 × 266 × 180 mm box, the foxtail millets were poured into a measuring cylinder to measure the volume, and the cooled bread was placed in each, filled with foxtail millets again, and the removed foxtail millets were placed in the measuring cylinder to measure the volume. The volume of the bread divided by the weight is the specific volume (mL/g):

(4) $$Specific\;volume(mL/g)=\frac{Loaf\;volume(mL)}{Weight\;of\;loaf(g)}\;$$

#### 2.2.7. Measurement of the Weight and Baking Loss Rate of Bread

The control group made with a fixed baking time and the experimental group made using BPPM were weighed, and the baking loss rate of the bread was expressed using the difference between the weight of the bread before and after baking as follows:

(5) $$Baking\;loss\;rate\left(\%\right)=\frac{Dough\;weight\left(g\right)-weight\;after\;baking(g)}{Dough\;weight(g)}\times 100$$

#### 2.2.8. Measurement of the Color of Bread

The color of the control group crust made with fixed baking time and the experimental group crust made using BPPM were measured in triplicate for lightness (L), redness (a), and yellowness (b) using a colorimeter (TES-135A, TES Electrical Electronic Corp., Taipei, Taiwan). The L-value of the standard color plate was 96.69, the a-value was 3.945, and the b-value was −0.632.

#### 2.2.9. SPGV

The camera configured in the SDMD measures and stores SPGV data inside the dough tub of the bread maker in real time. To extract the dough image inside the dough tub, the camera frame is set to 275 × 100, and averaging blurring is implemented using the blur library provided by OpenCV to minimize noise in the extracted image. During the baking process, the grayscale value of every pixel of each image, which is stored continuously in communication with the workstation, is summed up to measure the SPGV. The SPGV was defined by the following equation:

(6) $$\sum_{i=1}^{n}\sum_{j=1}^{n}G_{i,j}=G_{1,1}+G_{1,2}+\cdots +G_{i,j}+\cdots +G_{n,n}$$

- *n* is the image size, *i* is the row index, *j* is the column index, and *G* is the grayscale value.

#### 2.2.10. Statistical Analysis

The data obtained as a result of this study were used to calculate ‘mean ± standard deviation’. All experiments were performed in triplicate, and significant differences between control and experimental group were analyzed by unpaired Student’s *t*-test and one-way ANOVA using RStudio (Version 4.3.1) program. The significance test after one-way ANOVA was performed by Duncan’s multiple range test at the *p* < 0.05 level to verify the significant difference between each sample.

## 3. Results and Discussion

### 3.1. BPPM

BPPM learning results are as follows. FSPM consists of a training dataset consisting of a total of 15,335 samples and achieved a learning accuracy of 0.9962 and a loss of 0.0097. FSPM achieved a validation accuracy of 0.9958 and a validation loss of 0.0114 using a test dataset consisting of 3834 samples. BSPM consists of a training dataset consisting of a total of 11,618 samples and achieved a learning accuracy of 0.9896 and a loss of 0.0254. BPPM achieved a validation accuracy of 0.9886 and a validation loss of 0.0303 using a test dataset consisting of 2905 samples.

**Figure 3:**
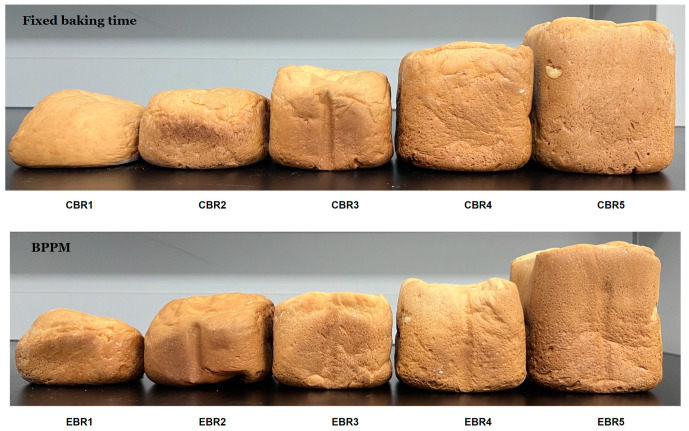
Appearance of bread made with fixed baking time and bread made using BPPM.

The results of applying BPPM are as follows. The appearance of the control bread made with fixed baking time and the experimental bread made using BPPM are shown in Figure 3. The time required to complete fermentation for experimental group EBR1-5 was 56.74 ± 2.56 min, 62.82 ± 4.85 min, 67.83 ± 2.60 min, 73.08 ± 4.64 min, and 76.92 ± 2.05 min, respectively, and the time required to complete baking was 41. 75 ± 3.12 min, 46.37 ± 2.37 min, 53.47 ± 0.89 min, 55.26 ± 5.34 min, and 61.66 ± 2.17 min, respectively, indicating that the baking process was completed somewhat later compared to the overall baking time of the control group.

### 3.2. Volume and Specific Volume

The results of volume and specific volume measurements of the control group made with fixed baking time and the experimental group made using BPPM are shown in Table 3. Both the volume and specific volume of the experimental group made using BBPM showed significant differences (*p* < 0.05) when compared to the control group. The volume (mL) of the control group was 112.44 ± 3.49, 387.78 ± 2.31, 636.57 ± 4.47, 823.87 ± 3.57, and 1198.81 ± 5.45 for CBR1-5, respectively, and the specific volume (mL/g) was 0.55 ± 0.03, 1.45 ± 0.04, 1.85 ± 0.00, 2.04 ± 0.03, and 2.50 ± 0.05, respectively. The volume of the experimental group was 127.54 ± 2.54, 413.49 ± 2.59, 679.96 ± 1.90, 875.79 ± 2.46, and 1260.70 ± 3.13 for EBR1-5, respectively, and the specific volume was 0.64 ± 0.01, 1.65 ± 0.02, 2.06 ± 0.01, 2.25 ± 0.01, and 2.69 ± 0.01, respectively, which showed a significant increase in volume and specific volume compared with the control group (*p* < 0.05). These measurements were consistent with the results of a study that reported an increase in bread volume with increasing fermentation time and yeast content [28].

**Figure 4:**
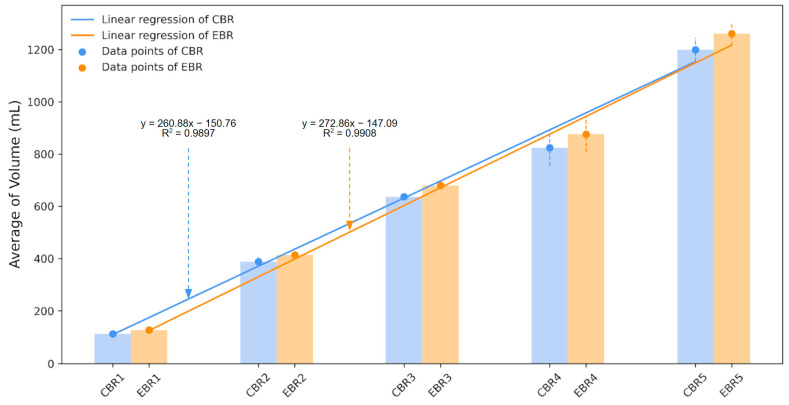
Linear regression graph of the average volume of bread made with a fixed baking time vs. bread made using BPPM.

The graph of the linear regression analysis for the comparison of the volume increase rate of the control group and the experimental group is shown in Figure 4. The results of the linear regression analysis of the control group showed that the volume increase slope was 260.88, the intercept was 150.76, and the volume increase slope of the experimental group was 272.86, and the intercept was 147.09. The results of the linear regression analysis showed a strong positive linear relationship between the variables x and y, with R^2^ values above 0.9 each, indicating high predictive reliability of the trend line. As a result, the volume increase rate of the experimental group made using BPPM was higher than that of the control group, which was statistically significant (*p* < 0.05).

### 3.3. Weight and Baking Loss Rate

The results of the weight and baking loss rate of the control group made with fixed baking time and the experimental group made with BPPM are shown in Table 4. Compared with the control group, the weight and baking loss rate of the experimental group made using BBPM showed significant differences (*p* < 0.05) except for CBR4 and EBR4. The weight (g) of the control group was 205.68 ± 3.06, 267.14 ± 5.92, 343.60 ± 2.28, and 479.32 ± 8.38 for CBR1-3 and CBR5, respectively, and the baking loss rate (%) calculated as Equation (5) was 23.61 ± 1.14, 25.59 ± 1.64, 23.43 ± 0.51, and 24.50 ± 0.46, respectively. The weight of the experimental group was 199.91 ± 1.58, 264.46 ± 1.07, 338.17 ± 1.62, and 468.75 ± 1.50 for EBR1-3 and EBR5, respectively, and the baking loss rate was 25.75 ± 0.58, 26.33 ± 0.30, 24.64 ± 0.36, and 25.39 ± 0.24, respectively, which showed a decrease in weight and an increase in baking loss rate compared with the control group (*p* < 0.05). These results were similar to the findings of Bosmans et al. (2013) [29] in that baking time and storage temperature induce changes in starch and gluten fraction, affecting the cursing process and moisture content of the bread, and for this reason, it is estimated that the weight of BPPM bread made with a relatively long baking time is low. It is believed that the weight of the bread decreased due to increased moisture loss. The baking loss rate is due to the volatilization of volatiles in the fermentation products and the evaporation of water by heating, and the water absorption capacity of the sample can affect the baking loss rate [30].

### 3.4. Color Analysis

The results of measuring the color of the control group’s crust made with fixed baking time and the experimental group’s crust made using BPPM are shown in Table 5. The L-value, which represents the brightness of the crust, showed that CBR1 and EBR1 had the highest values of 41.13 and 39.59, respectively, and EBR5 and CBR5 had the lowest values of 31.03 and 34.40, respectively, which were statistically significantly different from the other samples (*p* < 0.001). These measurement results were similar to the study results of Içöz et al. (2004) [31], which showed that the L-value, a measure of brightness, decreased and the color of the bread darkened as the baking time and temperature increased, and for this reason, the L-value of the bread decreased with increasingly longer baking times for each loaf. For the a-value, which represents redness, CBR5 and EBR4 showed the highest values of 16.66 and 15.32, respectively, while EBR1 and CBR1 showed the lowest values of 12.01 and 13.50, respectively, with significant differences from other samples (*p* < 0.001). The b-value, which represents yellowness, was the highest for CBR1 and EBR1 at 26.99 and 26.77, respectively, like the L value, and the lowest for CBR5 and EBR5 at 19.04 and 19.14, respectively, showing a significant difference from other samples (*p* < 0.001).

**Figure 5:**
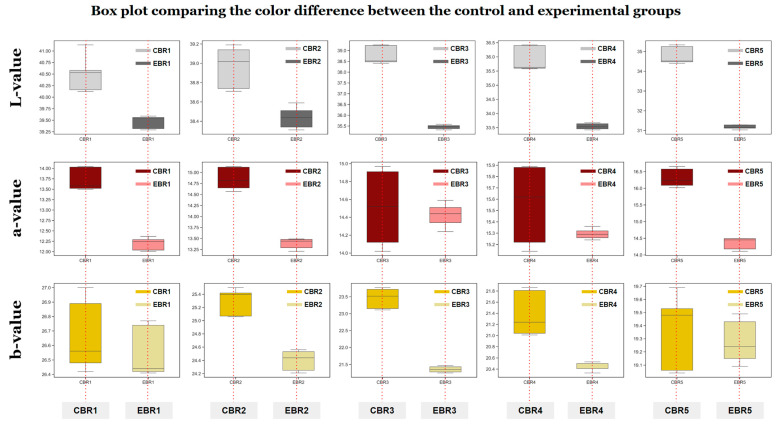
Box plot comparing the average volume of the control group to the average volume of the experimental group.

The visual comparison of the color of the control and experimental group is shown in Figure 5 using a box plot. The box plot analysis showed that the experimental group had lower maximum, minimum, and median values and smaller ranges for the first quartile (Q1) and third quartile (Q3) than the control group. These results suggest that the color of the experimental group made using BPPM is consistent.

### 3.5. SPGV

**Figure 6:**
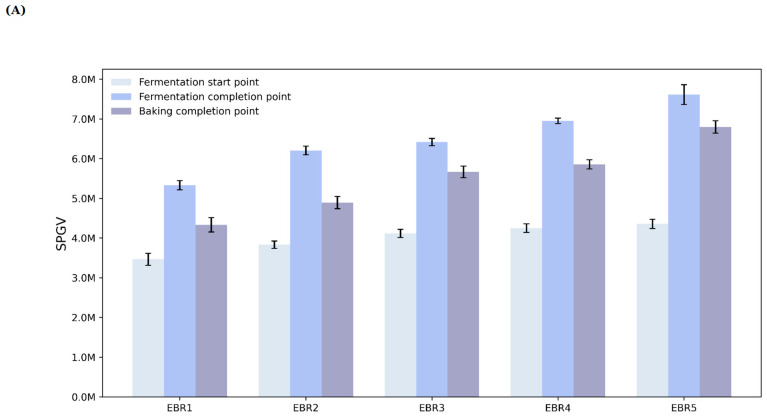

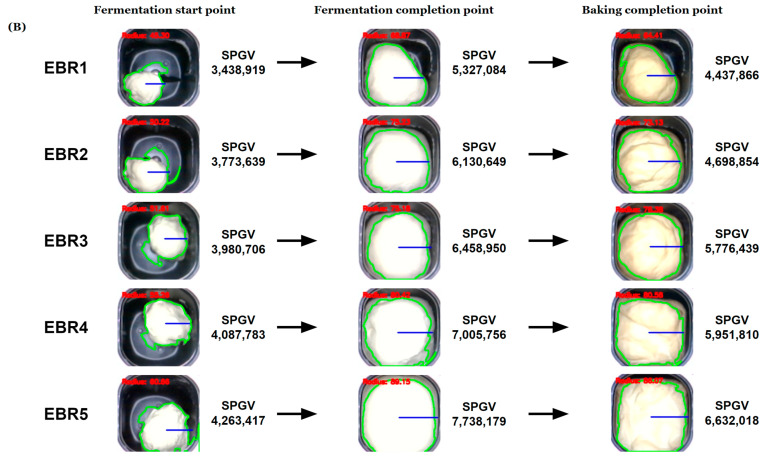
(**A**) Changes in SPGV for each point of bread made using BPPM. (**B**) Changes in SPGV due to dough size and color. The green-colored line represents the boundary of the dough volume.

The variation of SPGV at each point of fermentation start, completion, and baking completion for the bread made using BPPM is shown in Figure 6A. The SPGV of the bread at the fermentation start point was highest for EBR5 at 4,434,234, followed by EBR4 (4,324,912), EBR3 (4,228,421), EBR2 (3,927,242), and EBR1 (3,632,942). This is due to the increase in dough volume during fermentation, as shown in Figure 6B, where the dough with a relatively high SPGV occupies a larger area of the dough tub with a lower SPGV, resulting in a lower overall SPGV (*p* < 0.05). At the fermentation completion point, EBR5 had the highest SPGV of 7,769,473, followed by EBR4 (7,023,285), EBR3 (6,503,829), EBR2 (6,320,573), and EBR1 (5,449,102), which is similar to the gradually increasing SPGV change from the fermentation start point. The SPGV of the bread at the baking completion point was highest for EBR5 at 6,934,823, followed by EBR4 (5,923,742), EBR3 (5,824,712), EBR2 (5,024,821), and EBR1 (4,442,412). Overall, the SPGV at the baking completion point was lower than the SPGV at the fermentation completion point, which may be due to the loss of moisture content and browning of the bread surface as baking process due to the caramelization and Maillard reaction occurring during baking, as reported by Capuano et al. (2008) [32] (*p* < 0.05).

## 4. Conclusions

In this study, the quality characteristics of bread made with fixed baking time and bread made using data-based BPPM trained with sensor and vision data were compared and evaluated, and the results of model training and application were evaluated. Compared to the control group, the volume of the experimental group showed a higher value, and the slope of the increase in volume was higher in the experimental group, and the specific volume was also higher in the experimental group. The weight was lower in the experimental group compared to the control group, and the baking loss rate was higher in the experimental group. Color measurements showed that the L and b-values increased with baking time for both the control and experimental groups, while the a-value tended to decrease. The box plot analysis also showed that the maximum, minimum, and median values were lower than the control group, and the Q1 and Q3 values were also lower. The appearance of the bread was similar to that of the bread with the same proportions of each ingredient, and the baking time required was later for the bread made using BPPM than for the bread made with the fixed baking time. The SPGV analysis, which was monitored and stored in real time during the baking stage prediction process, showed that the SPGV data increased with the baking process. In the future, it is expected that sensors with higher measurement sensitivity will be used to predict the baking process more accurately. If a large dataset of various recipes is trained, it is expected to be able to predict the desired baking stages and produce uniform, quality bread even when the user uses the desired amount of ingredients and additives. The results of this study are expected to lay the foundation for the future development of AI and ML and the food industry.

## Figures and Tables

**Table 1:** Ingredient formula of breads (unit: g).

Ingredient	Samples (%)				
	CBR1 ^(1)^	CBR2	CBR3	CBR4	CBR5
	EBR1 ^(2)^	EBR2	EBR3	EBR4	EBR5
Time (CBR1-5)	Kneading mode/Fermentation mode/Baking mode (min)				
	7.5/55/40	10.0/60/45	12.5/65/50	15.0/70/55	17.5/75/60
Time (EBR1-5)	AI	AI	AI	AI	AI
Strong flour	100	133.33	166.67	200	233.33
Water	60	60	60	60	60
Yeast	2	2	2	2	2
Sugar	10	10	10	10	10
Cooking oil	7.5	7.5	7.5	7.5	7.5

^(1)^ CBR1: Strong flour 100%. ^(2)^ EBR1: Strong flour 100%. The control group is CBR1-5, and the experimental group is EBR1-5.

**Table 2:** Specifications of the sensors composed in SDMD.

Sensor	Measurement	Sensitivity
MAX6675	Temperature	0–1024 °C
MQ3	Ethanol	0.05–10 mg/L
GP2Y0A02YK0F	Distance	20–150 cm
ELP-USBFHD08S-MFV	RGB, Radius, SPGV	260 fps

**Table 3:** Volume and specific volume of bread made using fixed baking time and bread made using BPPM.

Samples	Volume (mL)	Specific Volume (mL/g)
CBR1 ^(1)^	112.44± 3.49	0.55 ± 0.03
EBR1	127.54 ± 2.54	0.64 ± 0.01
*t*-value	−6.05 **	−4.64 **
CBR2	387.78 ± 2.31	1.45 ± 0.04
EBR2	413.49 ± 2.59	1.65 ± 0.02
*t*-value	−12.83 ***	−8.20 **
CBR3	636.57± 4.47	1.85 ± 0.00
EBR3	679.96 ± 1.90	2.06 ± 0.01
*t*-value	−15.46 ***	−32.00 ***
CBR4	823.87 ± 3.57	2.04 ± 0.03
EBR4	875.79 ± 2.46	2.25 ± 0.01
*t*-value	−20.73 ***	−13.14 ***
CBR5	1198.81 ± 5.45	2.50 ± 0.05
EBR5	1260.70 ± 3.13	2.69 ± 0.01
*t*-value	−17.07 ***	−6.11 **

^(1)^ Refer to Table 1. ** *p* < 0.01; *** *p* < 0.001. Data were compared with Student’s *t*-test (*p* < 0.05).

**Table 4:** Weight and baking loss rate of bread made with fixed baking time and bread made using BPPM.

Samples	Weight (g)	Baking Loss Rate (%)
CBR1 ^(1)^	205.68 ± 3.06	23.61 ± 1.14
EBR1	199.91 ± 1.58	25.75 ± 0.58
*t*-value	2.91 *	−2.90 *
CBR2	267.14 ± 5.92	25.59 ± 1.64
EBR2	264.46 ± 1.07	26.33 ± 0.30
*t*-value	0.77 *	−0.77 *
CBR3	343.60 ± 2.28	23.43 ± 0.51
EBR3	338.17 ± 1.62	24.64 ± 0.36
*t*-value	3.36 *	−3.35 *
CBR4	403.45 ± 5.44	25.08 ± 1.01
EBR4	396.66 ± 0.99	26.34 ± 0.19
*t*-value	2.13 ^NS^	−2.12 ^NS^
CBR5	479.32 ± 8.38	24.50 ± 0.46
EBR5	468.75 ± 1.50	25.39 ± 0.24
*t*-value	2.94 *	−2.94 *

^(1)^ Refer to Table 1. * *p* < 0.05; ^NS^ not significant. Data were compared with Student’s *t*-test (*p* < 0.05).

**Table 5:** Color values of bread crust made using fixed baking time and bread crust made using BPPM.

Samples	L	a	b
CBR1 ^(1)^	40.50 ± 0.40 ^a^	13.72 ± 0.25 ^e^	26.66 ± 0.23 ^a^
CBR2	38.97 ± 0.20 ^b^	14.86 ± 0.23 ^c^	25.29 ± 0.18 ^b^
CBR3	38.74 ± 0.39 ^b^	14.51 ± 0.38 ^d^	23.47 ± 0.27 ^c^
CBR4	35.88 ± 0.40 ^c^	15.56 ± 0.31 ^b^	21.36 ± 0.36 ^d^
CBR5	34.75 ± 0.41 ^d^	16.30 ± 0.25 ^a^	19.37 ± 0.26 ^e^
*F*-value	375.30 ***	105.60 ***	1066 ***
EBR1	39.48 ± 0.13 ^a^	12.20 ± 0.14 ^d^	26.54 ± 0.16 ^a^
EBR2	38.44 ± 0.11 ^b^	13.38 ± 0.11 ^c^	24.40 ± 0.14 ^b^
EBR3	35.45 ± 0.09 ^c^	14.42 ± 0.12 ^b^	21.35 ± 0.08 ^c^
EBR4	33.47 ± 0.10 ^d^	15.29 ± 0.04 ^a^	20.43 ± 0.07 ^d^
EBR5	31.17 ± 0.10 ^e^	14.36 ± 0.17 ^b^	19.30 ± 0.15 ^e^
*F*-value	9526 ***	823.1 ***	4879 ***

^(1)^ Refer to Table 1. *** *p* < 0.001. ^a~e^ Means denoted in a column by the same letter are not significantly different (*p* < 0.05).

## Data Availability

The data used to support the findings of this study can be made available by the corresponding author upon request.
